# Psychological Health and Life Experiences of Pregnant Adolescent Mothers in Jamaica

**DOI:** 10.3390/ijerph110504729

**Published:** 2014-04-30

**Authors:** Karline Wilson-Mitchell, Joanna Bennett, Rosain Stennett

**Affiliations:** 1 Midwifery Education Program, Ryerson University, 350 Victoria Street, Toronto, ON M5B 2K3, Canada; 2 The UWI School of Nursing, The University of the West Indies, 9 Gibraltar Camp Way, Mona, Kingston 7, Jamaica; E-Mails: joanna.bennett03@uwimona.edu.jm (J.B.); rosain.stennett@uwimona.edu.jm (R.S.)

**Keywords:** adolescent, pregnancy, psychosocial distress, abuse, resilience, antenatal care, social support

## Abstract

A recent Jamaican school-based survey revealed that 23.1% of 13–15 year-olds, had attempted suicide one or more times during the last 12 months. Research that links adolescent pregnancy and suicidal behaviour is lacking in Jamaica. Psychological distress and suicidal behaviours amongst pregnant adolescents elsewhere in the Americas has been documented at prevalence of between 13.3%–20%. The purpose of the study was to explore the experiences and the impact of pregnancy on pregnant adolescent psychological health. Individual interviews and focus groups were conducted with adolescents in two Jamaican antenatal clinics. One clinic was designed as a ‘Teen Pregnancy Clinic’ and the other used the standard antenatal clinic design. The following themes were identified: decision-making, resilience, social support, community support system, distress, and perceptions of service. Participants reported positively on the specific interventions tailored to their needs at the Teen Clinic. Although motherhood is valued, none of the pregnancies in this study were planned by the mother. Of the 30 adolescents interviewed, seven cases were referred for counseling due to their need for emotional and psychological support. One of the adolescents reported recent sexual violence and another reported having experienced childhood sexual abuse. Historically, Jamaican adolescent mothers faced barriers to education, self determination, and family planning. Empowering, adolescent-centred healthcare and comprehensive reproductive health education may mitigate psychosocial distress.

## 1. Introduction

The 2008 Jamaica Reproductive Health Survey (RHS) has shown a decline in adolescent (females 15–19 years) pregnancy from 137 in 1975 to 79 in 2002 and 72 in 2008 [1]. Despite this downward trend, there remains significant concern about the high rate of adolescent pregnancy and the subsequent negative impact, including high maternal mortality, premature babies, complications during labour, low birth weight rate, low and poor utilization of antenatal health care [1]. Maharaj *et al.* [2] reported a 15–20% prevalence rate for adolescent pregnancy in Jamaica, with one-fifth of these in their second pregnancy. Throughout this article, participants will be referred to as pregnant teenagers or adolescent mothers. This conceptualization recognizes the fluid and dynamic transition to motherhood that begins prior to birth; which is in keeping with current literature [3,4].

The authors shared a concern regarding the psychosocial impact of adolescent pregnancy, including interruption in education and the attainment of individual goals and lack of parental support at a time when they are emotionally and financially vulnerable. Additionally, the transition to motherhood is reported to put adolescents at a greater risk for psychological distress. Adolescent mothers have been shown to be more likely to present with symptoms of depression when compared with their non-parenting peers and older mothers [5].

Additional evidence of psychological distress among Brazilian pregnant adolescents has been reported by Freitas *et al.* [6], who conducted a case-controlled study to compare the psychosocial profile and suicidal behaviour of 110 pregnant and 110 non-pregnant adolescents. The findings indicated that adolescent pregnancies were associated with: substance use, low level of social support, depression, traumatic life events and other psychosocial difficulties. The study found pregnant adolescents had higher prevalence rates than non-pregnant girls, for attempted suicide (20% *vs.* 6.3%), depression (26.3% *vs.* 13.6%) and anxiety (43.6% *vs.* 28%).

These findings are further supported by a cross-sectional study of 828 pregnant teenagers conducted in southern Brazil by Pinheiro *et al.* [7]. They found that suicidal behaviour or intentional deaths or attempts to take one’s life, were relatively common features in pregnant teenagers. Prevalence of suicidal behaviour was 13.3%, with 1.3% reporting attempting suicide within the last month. Further significant associations were found between suicidal behaviour and low education, prior abortion, previous major depression, and physical abuse within the last 12months. Pregnant teenagers with high social support showed prevalence ratios 67% lower than those with low social support. A wide range of psychiatric disorders, most notably major depressive disorder and panic disorder remained associated with suicidal behaviour after adjustment. Moreover, Mollborn and Morningstar [8] found that teenage mothers had higher levels of psychological distress than their childless adolescent peers and adult mothers. They suggest that the experience of teenage childbearing did not appear to be the cause, as teenage mothers’ distress levels were already higher than their peers before they became pregnant.

The literature concerning Jamaican adolescents primarily describes negative experiences such as substance abuse, unplanned pregnancy, sexually transmitted infections, violence, and crime [9,10,11]. There is a paucity of research that examines positive attributes such as self esteem or resilience in Jamaican adolescents. Furthermore, there have been few studies to explore the factors that influence the mental health status of pregnant adolescents. The current study reflects a framework that incorporates strengths, potential, resilience and mental health. Antonovsky’s Sense of Coherence (SOC) Scale draws connections between mental health, resilience and level of coherence [12]. Antonovsky’s scale seems to measure the process by which individuals perceive stressful situations and “use general resistance resources to maintain and develop their health” [12]. This tool has been used in over 30 languages and countries, with various children and adults and finds applicability irrespective of culture. The SOC scale has accurately predicted social and mental health outcomes. Another model, The Positive Youth Development (PYD) model, similarly provides a multidimensional approach using 40 assets that are identified as important components of adolescent mental health and socio-economic success [13]. Although they address various social determinants of health, neither the SOC nor the PYD model has incorporated critical race/class/child rights issues that influence post-colonial Jamaican society. And it is unknown whether these models are sufficient to explain the responses of Jamaican adolescent mothers to pregnancy; however, these frameworks have informed this project and the conceptualization of mental health that evolved from the study findings. 

A study of this nature is relevant within the Jamaican context in light of public health concerns arising from the increasing rate of suicidal ideation and behaviour among adolescents. The Jamaica Youth Risk and Resiliency Behavior Survey 2005 noted that 12% of female adolescents 10–15 years old experienced suicidal ideation [10]. Since that time, the 2010 School-based study noted that 25.7% of females 13–15 years old experience suicidal ideation and 23.1% of them had attempted suicide one or more times during the preceding 12 months [14]. Additionally, there is evidence indicating that Jamaican pregnant adolescents experience feelings of sadness, shame and emotional turmoil when they find out about their pregnancy [15]. Further it has been reported that pregnant teenagers in Jamaica report feeling stressed, depressed and often experience stigma surrounding marital status and ageism [16]. There are anecdotal accounts of desperate, pregnant adolescent teens seeking unsafe, illegal abortions [16]. Based upon these concerns expressed in the media and the international evidence linking suicidal ideation and behaviour to adolescent pregnancy, it is prudent to examine this association in Jamaica.

This study, which explores the self-reported perceptions of pregnant adolescents, seeks to provide interim data which could inform the development of larger scale studies that focus policy and practice in relation to adolescent reproductive and maternity healthcare. The purpose of the study was to describe the socio-demographic profile and to explore the personal experiences of pregnant adolescents and the impact of pregnancy on Jamaican adolescents’ psychological health. In addition, comparisons were made between the mothers’ experiences of healthcare received in the teen-centred clinic compared to a standard antenatal clinic.

## 2. Methods

Mixed methodology was employed to identify psychosocial factors associated with adolescent pregnancy and suicidal risk. Semi-structured interviews and guided focus group discussions were utilized in collecting data. 

### 2.1. Participants and Sampling Methods

Convenience sampling methods were used to recruit adolescents ages 12–17 years old who visited the antenatal clinics at two urban hospitals in Jamaica. Hospital A held a “Teen-Centred” clinic and Hospital B held a standard antenatal clinic. The participants were solicited verbally by the two trained research assistants who presented in the clinics during the weeks preceding and during the data collection. Adolescents were excluded from the study if they were diagnosed with a learning disability, or were unable to speak, read or write. Individual consents were obtained for adolescents 16 years and older. Assents as well as parental consents were obtained when participants were under the age of 16. 

The sample consisted of 30 pregnant adolescents, 15 from each antenatal clinic. The focus group sample consisted of 16 pregnant adolescent, eight from each hospital. Only two of those who participated in the interviews agreed to participate in the focus group discussion. The focus group discussion and semi-structured interviews were conducted in a quiet space at both hospitals. Refreshments were provided for the participants along with a department store and supermarket coupons worth $1000 JAD (approx. $10 USD) with which they could purchase items in preparation for their babies. Focus group preparation and facilitation utilized well established protocols described by Morgan [17,18].

Semi-structured interviews included questions which provided both quantitative and qualitative data. The findings presented in this paper will report solely on the preliminary analysis of the individual interviews. Future papers will present comparative findings of the analysis of the focus group discussions from both hospitals.

### 2.2. Data Collection

The risk of harmful emotional distress during data collection was minimized by providing immediate referral to the senior midwife to initiate the referral process for counseling. The two participants that reported sexual violence and abuse were referred to the Child Development Agency as mandated by the Jamaican Child and Protection Act [19]. Interview appointments were made immediately prior to or following regularly scheduled clinic visits so as not to be disruptive. Participants were free to decline an answer, to stop or to leave individual or focus group interviews at any time if there was a feeling of discomfort with the process or questions. Audio recording of interviews and group discussion was optional. All of the participants accepted for the study consented to audio recording. One of the respondents declined audio recording and was excluded from interviews. None of the participants requested an early exit from interviews or focus groups. The potential participants were screened by the nursing staff to avoid parental consent of adolescents who were potentially in abusive or coercive situations. One adolescent was interviewed who self identified for the first time as a survivor of sexual violence from an unknown assailant. The assault resulted in her present pregnancy and so she was a candidate for protective custody of a foster parent or the services of a Children’s Advocate. Only adolescents in home situations which were deemed “safe” by the clinic staff were approached. Safety was defined as an absence of verbal or physical abuse [19]. Ethics Board approval was obtained from University of the West Indies, Ryerson University, the Ministry of Health, Jamaica and the South East Regional Health Authority, Jamaica. Quantitative methods were employed to obtain descriptive statistics of the sample of 30 individual interview participants.

### 2.3. Questionnaire

The questionnaire and discussion tool were designed to elicit themes regarding perceptions, values, resilience, knowledge of community resources, perceptions of social support, sexuality, reproductive health knowledge and psychological distress during a 1 hour interview. A pilot was performed with four participants. This provided valuable feedback in order to modify the individual questionnaire language to elicit more discussion and to improve clarity. The final individual questionnaire held 41 guiding questions divided into the following categories: demographics, conception and reproductive health, current pregnancy, relationship and sexuality, antenatal clinic service, depressive symptoms, resilience or protective factors. The 1 hour focus group guide contained six possible guiding questions: (1) experiences with family, partner, community and school, (2) symptoms and feelings about pregnancy, (3) dreams and hopes for the future, (4) barriers to achieving dreams and hopes, (5) facilitators to reaching goals, (6) self-identified personal strengths which support coping. Both researchers and assistants were well versed in the Jamaican patois dialect which expedited the analysis and interpretation of questionnaire responses. The four initial pilot interviews were not included in the study analysis. Analysis of the focus group discussions will be reported elsewhere.

### 2.4. Statistical Analysis

Descriptive statistics were used to describe the sample of 30 individual interview participants using SPSS. Thematic analysis using grounded theory methods [20,21] was used for the 30 individual interviews and two focus group interviews. Preliminary themes were reviewed and compared within the research team using NVIVO-10 and re-categorization and recoding followed with in-depth analysis of language and concept mapping [20,21]. 

## 3. Results

### 3.1. Sample Demographics

Following are demographic descriptions of the adolescent mothers that were interviewed in both hospitals (See Table 1). The two groups were compared in terms of age, parity, gestational age at interview, obstetrical complications, smoking or substance use, type of dwelling, individuals with whom they cohabited, and continuance of schooling. All of the pregnant adolescents were primigravidas (experiencing their first pregnancy). The majority (26.6%) of them were interviewed at “7 months” pregnant (approximately 28–30 weeks gestation). At the time of conception, school attendance for the Hospital A pregnant adolescent was 93.3% and for the Hospital B was 87.7%. Following the discovery of pregnancy and the commencement of antenatal care, only 46.7% of the Hospital A pregnant adolescents continued their education while only 40% of the Hospital B pregnant adolescent continued their education. In other words, 57% of the 30 pregnant adolescent discontinued their education due to the pregnancy. 

**Table 1:** Demographic characteristics of the pregnant teenagers.

Demographics	Total Population N = 30	Hospital A N = 15	Hospital B N = 15
**Mean Age in Years**	15.6 (SD 0.932)	15.6 (SD 0.507)	15.6 (SD 1.242)
**Parity (Previous Births)**	0	0	0
**Smoking/Substance Use**	60% (18)	53.3% (8)	66.7% (10)
**Type of Dwelling:**			
Family Home	53.3% (16)	60% (9)	46.7% (7)
Owner Occupied	36.7% (11)	33.3% (5)	40% (6)
Renting	10% (3)	6.7% (1)	13.3% (2)
**Cohabitants:**			
Mother	60% (18)	60% (9)	60% (9)
Parents	13.3% (4)	13.3% (2)	13.3% (2)
Grandmother	13.3% (4)	13.3% (2)	13.3% (2)
Aunt	6.7% (2)	6.7% (1)	6.7% (1)
Sibling	3.3% (1)	0% (0)	6.7% (1)
Other	3.3% (1)	6.7% (1)	0% (0)
**School Attendance at Conception**	90% (27)	93.3% (14)	86.7% (13)
**Continuance of School Attendance **	43.3% (13)	46.7% (7)	40% (6)
**Boyfriend Involved at Time of Interview**	60% (18)	73.3% (11)	46.7% (7)

Other questions were asked to elicit social and obstetrical factors. Two teenagers described significant medical conditions (thyroid disease and seizure disorder). Participants were asked about substance abuse, relationship status, history of intimate partner violence or child abuse, financial supports, decision-making, gestational age at the time of interview and presence of any health complications. When asked about experimental or recreational use of drugs, 60% of the mothers responded in the affirmative, however 81.3% of them stopped with the diagnosis of pregnancy. Only 60% of the pregnant adolescents reported currently having a “boyfriend”. The majority of the boyfriends were 19 years old or older (75.7%). Although many of these relationships had lasted 3–4 years starting during the boyfriend’s adolescence, they continued with an adolescent female of less than 17 years of age. When asked about continuity of the relationship following pregnancy, 28.6% of the pregnant adolescents reported that the relationship was not sustained, 67.9% reported that the relationship continued, and 3.6% (one mother) reported “sometimes”. When asked about sexuality, 93.1% (27 mothers) reported that their partner did not “pressure them to have sex”, 60.7% reported that they did not plan to have intercourse with their partner, and 20% (six mothers) reported that they had experienced “forced sex” in their lifetime. When specifically asked if an adult had ever “touched you in a sexual way”, 17.9% (five mothers) reported “yes”. When asked if they had provided sex to “assist the family financially”, 10.3% (three mothers) responded “yes”.

In most cases (36.7%) the pregnant adolescents reported informing a “family member first”; 26.7% of the time the partner was informed first; and 16.7% of the time a friend was told first. In 20% of the cases “no one” was informed of the pregnancy. Participants were not always sure how they wanted to proceed with the pregnancy. A slightly greater numbers of mothers (16) were unsure of what they wanted to do about pregnancy. Mothers usually relied on a family member (46.5%) to assist them with the decision of how to proceed with pregnancy; while in 21.4% of the cases, the mother reported that no one helped her to make the decision. Elective termination of pregnancy is illegal currently in Jamaica.

### 3.2. Thematic Analysis

Although thematic analysis was framed by a resilience perspective the data revealed findings of great concern to the researchers. Seven out of the 30 participants (23%) interviewed experienced psychological distress and were referred for psychological support. Two of the adolescent mothers (6.6%) exhibited suicidal ideation or entertained thoughts of killing themselves. This was not a predominant theme within the analysis, however the risk of such a high degree of psychological distress existing amongst female adolescents warrants further investigation. Consequently the themes described represent dimensions of adolescent motherhood that denote variables on a continuum that may indicate positive or negative attributes from the perspective of the mothers.

Because of the Jamaican vernacular, word and concept queries using the NVIVO data management system were challenging (e.g., “dem a pree mi” means “they are looking at me”. “Good for you!” is meant as a sarcastic remark and not a supportive statement.). Nonetheless, code density was reached on several common experiences and concepts. These preliminary themes included the following: 1. Perceptions of service; 2. Resilience; 3. Decision Making; 4. Social Support; 5. Community Support System; 6. Distress versus Psychosocial health. Following are examples of quotations in italics that illustrate the respective themes (words are bolded for emphasis).

1. **Perception of Services:** The participants provided deep insights into their perceptions of the institutional responses to the issues of adolescent pregnancy. In some cases they indicated that the staff demonstrated encouragement, caring and concern.

“*I like the fact that as you start coming to the clinic as a teenager, yes they introduce you to go back to school... and they always make sure they talk to you (about) what to eat just to keep your baby all of that stuff, to take care of the baby, don’t be stress about it*” *(A-R1)*

“*Looking out for us like my age...because every second they call and make sure that we are sitting and we are listening and we are okay” (B-R3)*

For the most part, the reactions of the adolescent to these interventions were positive since they prepared the adolescent for birth and motherhood.

“*They give **a lot of advice** (about) breast feeding, how to hold the baby, what should the baby get... how to treat the baby as well and what else, about hygiene and so forth, **they basically talk to us about everything***” *(A-R6)*

“*It’s okay they get along with me good **they ask questions that I need to hear**, and they also like to give us instructions what to do and all of that*” *(A-R8)*

There were, however, instances when their reactions were negative to the services received such as the lengthy waiting times following intake. These waiting times could average 4–6 h, during which time the non-clinical teachers visited to discuss nutrition, lactation, parenting or childbirth preparation.

“*(And what don’t you like most?) The long waiting to sit down and wait…. (how long?)… Sometimes when you come you have to sit down for a whole length of period of time.*” *(A-R5)*

“*Like when we are suppose to go and see the doctor they take too long to call my name…. Long hours, long time.*” *(A-U6)*

The reactions to the services rendered were tempered by the perceptions of the clinic environment and the reaction the adolescents received from other patients.

“*(Adult patients waiting in clinic) Staring (at me)…. It’s okay because they won’t be the first and they won’t be the last…. **They stare at you or criticize and all those things…. It makes me feel a way sometimes** but like I say I won’t be the first young person to get pregnant and I won’t be the last.*” *(A-R1)*

2. **Resilience:** The interviews revealed several strengths possessed by the adolescent mothers. The most notable characteristic was resilience. Resilience was closely tied to goal-oriented speech.

“***I am coping with everything right now** just that I need a little more support financially just that… **Whenever something knocks you down you have just to get back up on your feet, and that is what I am planning to do**…Yes something knocks me down just stand to rise and I am going to arise even stronger (**Interviewer:** You think so?)…I know so! I am going to achieve them (goals that you have set)…. I like to finish school**, I tell myself say I want to be either a doctor or a midwife, so I am going to work very hard.***” *(A-R5)*

Resilience was also closely associated with a focus on the future and a motivation to “overcome”.

“*So **I will have to keep my mind steady on my plan going ahead of me** ... You have the baby so you can’t just take the baby out of the picture at all times, **but you have to go forward***” *(B-R7)*

“*Yes, worst I never plan for it so **I have to show that I can’t let this break me down***” *(A-R11)*

“*Like I say **being pregnant can be a set-back in your journey** sometimes, but it is not the end of the road. You **can still pick up and go again** and know that obstacles will come*” *(B-R9)*

“*Because at that time in your life you have a daughter and you don’t want her to follow in your foot steps for her to follow in your footsteps is a good way. Don’t make the same mistake that you made or live the lifestyle that you live. So those people want to set a good example like myself here*” *(A-R5)*

“*So **I will have to keep my mind steady on my plan going ahead of me** ... You have the baby so you can’t just take the baby out of the picture at all times, **but you have to go forward***” *(A-R4)*

3. **Decision-making:** One of the other aspects of character was the adolescent’s ability to make decisions. Sometimes the decision-making was influenced by her partner.

“*At first no I wasn’t sure **because I wanted to abort it at first …. Actually my boyfriend stop me say that it was his first child** and he is not sure if he is going to get a next one or I am going to be able to get pregnant in the future. So I just decided to bring it.*” *(A-R5)”*

At other times the family’s influence was the strongest.

“*All right **our family doesn’t believe in abortion so my only option was to have the baby** I didn’t want to have the baby ...**my grandmother she told me that is not right** and I was a Christian before*” *(B-U1)*

Finally, there were adolescent mothers whose decision-making was the culmination of a personal choice.

“*Very sure, yes **what can I do but keep it, this is our doing** so....Nobody (helped to make decision to continue pregnancy)*” *(A-R4)*

“*Well at first I wasn’t planning to use any contraceptive neither to throw away my baby or anything at all. **I really plan to keep my baby***” *(B-R8)*

4. **Social Support:** Another theme that was observed was that of “social support”. The support received from family, partners, and friends was diverse. Sometimes there was encouragement from significant others.

“*No she (Mom) didn’t say anything I expect her to cuss, **she always told me that if I got pregnant she would put me out and she wouldn’t be there for me she would be gone, So I am just surprise she is actually there right now**……but my dad he flips…For the first two months when my parents found out he (Dad) **stop preparing like food and those things,** when I was pregnancy I was still going to school, so he **stop leaving lunch money and those things.** So I actually have to find those things on my own as if my mom prepares those for me.*” *(A-R5)*

Sometimes the support was unexpectedly present and strong.

“*… But since my mother and other people talk to me and say I cannot stress out myself and like that, because I hide behind doors and cry a lot and a lot of the time my mom catch me, and say I must stop doing it, but **my mother say she will take the pressure**.*” *(B-R2)*

“*Daddy don’t say one thing (did not react negatively). Him always say well him can’t do nothing, **he said that he is glad to become a grandfather** before anything happen to him in life*” *(A-R14)*

5. **Meso/Macro-Community Support System:** Support from individuals was distinguished from support from the community at large. Systemic support based upon institutional policies and procedures or support that came about from the cultural mores or customs in the region provided significant material to formulate a separate theme. The educational policies and their implementation was a subject of contention and disappointment to many of the adolescent mothers.

“*But until then I was kicked out of school because the school found out about it. So I had to stop.*” *(A-R5)*

By contrast, the residential homes for wards of the state provided a welcomed supportive environment.

“*And plus the house mothers (at the Children’s Home)…. **If I feel depressed I go to them and talk to them, and I have a counselor**…Because my counselor like she say some people would find that you are pregnant they would give up on you, she say if they give up on you now when it is the time you need it, **she don’t see no reason why they should give up on you. She love us she talk good things about us**, she don’t keep anything that is bad.*” *(B-R5)*

6. **Distress *versus* Psychosocial health:** Psychological health was observed on a continuum ranging from good mental health to instances of marked psychological distress. Sometimes the distress accompanied evidence of unpreparedness for pregnancy.

“***Well I try to kill myself two times**….I take up a knife and I put it like this…(To your stomach two times?)…Yes Miss….(How long ago was that?)…. **When I find out…. I never want to continue (living)** Miss…I **can’t manage the baby!***” *(B-R2)*

“*Yes miss at first **when I found out every day I cry…Anytime I would talk about it, every day I would cry**… That is why I really don’t talk about it….(You don’t really like to talk about it, so you think maybe you could do with some counseling sometimes that’s help? Why you cry, what you think makes you cry?)….Because how my father felt and because I wasn’t ready…because (of how) some people feel and talked about it.*” *(B-R1)*

Sometimes psychosocial good health was evidenced by the ability to accept the reality of the situation.

“*I think so because it wasn’t expected, they fret I mean not fretting **but it is going to be on your mind** whether you like it or not. **It is going to be on the back of your head, it is going to be right there in front of you.** (Have you ever had any of those feelings - bad or sad or depressed or fret a lot when you found out you were pregnant?)…Yes, I said oh my God is this for real? You would be touching it…do you think it is something in there?*” *(A-R1)*

Finally, there was evidence that psychological health was closely related to the mother’s ability to “coach herself” into resilience. Many times the coaching of a support person (often the teen’s mother) was echoed in the words of the adolescent mother.

“*(Is this your first pregnancy?)….Yes and last!... No more I am not even sure if I am going to survive this first one! **That is no way on my agenda no, time soon! When I am set and ready in life maybe; or adoption but no never again.** I am positive and I am certain of that! …. It is true, Lord Jesus, people usual say one “pickney a nuh pickney” but this have to be the one, one, one. When I am ready and set in life when I am about thirty when I should have certain assets and things going for me, if I am marry or something or I am in a committed relationship maybe I will consider going again, especially if the person doesn’t have a child.*” *(A-R1)*

Although there were six preliminary themes analyzed and saturated, the researchers continued to interpret the data. No doubt future analysis on various levels will yield new and complex layers of subthemes such as: perceptions of self, values, other aspects of resilience, knowledge of community resources, perceptions of social support, psychological distress, powerlessness, and motherhood.

## 4. Discussion

The age range sampled in this study is in keeping with previous studies, with the majority of teen pregnancies occurring around age 15 years old and a smaller number of teen pregnancies occurring as young as 13 years old. Across the Caribbean and Latin America teen pregnancy rates are increasing. Currently two out of every 18 pregnant adolescents are 15 years old or younger [22]. 

Out of 30 participants, seven (23%) experienced psychological distress or suicidal ideation. This is consistent with the Brazilian research [6,7]. There were two reports of suicidal ideation (6.6%) where the adolescent mother mentioned the intention to kill herself. Although the sample size was too small to generalize these findings, this may indicate a growing problem. Further investigation of these cases may inform healthcare practice and prevention strategies by clinicians. The research assistants in our study were not permitted to make medical diagnoses but simply referred participants who demonstrated emotional distress or described self harm or abuse and so required and desired counseling. This rate was lower than the prevalence in Jamaican female adolescents noted in the 2005 and 2010 studies [10,14] and could be explored further in a nation-wide survey. Wilks’ study sampled only 15 students each from 18 schools (1320 students total), however findings revealed that female secondary school students (15–19 years or age) experienced more depressive symptoms their male counterparts [23]. Furthermore there was a prevalence of feelings of hopelessness (20.4%), feelings down/Depressed (41.3%), little interest/pleasure in activities (38.8%), change in appetite (42.9%), change in sleeping pattern (34.6), feeling guilty/worthless (14.8%), considered suicide (9.7%) attempted suicide (5%), or planned suicide (5.7%) [23]. Although these school-based surveys have very little exploration of pregnancy-related issues, 180 (25%) of the 721 female students were self identified as pregnant. Wilks noted that 65 (36%) of these pregnant teenagers experienced symptoms such as sadness or loneliness; 42.1% felt hopelessness; 36.5% reported “feeling depressed”; 39.4% reported feeling guilt in the past year and 45.1% of these same students who had been or were pregnant had also attempted suicide [23].

Antenatal distress could place adolescents at risk for postpartum depression amongst other illnesses and so it is reasonable for mental health services to be easily accessible in maternity clinics. It will be important for maternity care providers to expedite referrals and to maximize accessibility to mental health services of pregnant adolescents considering the high incidence of unplanned pregnancy and forced sexual contact described in the literature [10,23]. Multidiscipinary teams such as the one at the teen-centred clinic, which include social workers and mental health counselors, would be beneficial when planning maternity care for adolescents.

Violence against teenage women continues to be a theme in the adolescent mother’s narrative. Further research is warranted to explore any possible associations between the experience of violence and abuse upon the Jamaican adolescent’s participation and success in education throughout the lifespan. Out of the 30 mothers, two described some type of abuse within their life time. One of those instances resulted in the pregnancy and involved significant violence. This is consistent with the findings of the 2005 Jamaican school-based study that reported that sexual activity is forced in 1:5 of 15–19 years old [10]. Furthermore, the 2005 survey noted that 14.3% of these females had been physically abused, and 28.1% females had been victims of physical attacks [10]. The literature describes the phenomenon of impaired learning that follows violence [24,25]. The response to violence should include partnership between government and community stakeholders to enforce existing safety and protection policy, provide crisis counseling, and to sustain resources that optimize the educational trajectory for adolescent mothers.

Resilience language was observed indicating the significance of social support and possibly coaching by significant others. All of the teens identified their mothers as significant sources of social support yet most were unable to discuss sexuality or contraception with their mothers. Parent-adolescent relationships continue to be fraught with trust and boundary issues. These findings are consistent with previous research [26]. In addition to the support of parent-adolescent dialogue, this study may indicate a need to engage adolescents in guided peer support for reproductive health education. 

Over half (57%) of the sample discontinued their education. These mothers were not using the government funded support systems designed to enable pregnant adolescents to continue their education. Currently the office of the Prime Minister funds the Women’s Centre of Jamaica Foundation (WCJF), a government agency. The WCJF resources include education, childcare, counseling, vocational coaching and support for adolescent mothers who are 17 years old and under. When adolescents participated in the WCJF programs, the likelihood of contraception after the first birth increased and second pregnancy rate was below 2% [15]. In addition, the likelihood increased of high school completion, employment, and higher monthly income. Furthermore, the teens’ perception of themselves was more likely to be one of holding a higher socioeconomic status, belonging to social clubs and engaging in regular social activities. In a historical cohort study of 260 teen mothers (87 of whom were WCJF graduates), 25% (22 of 87) of participants were employed versus about 13% of comparisons (23 of 173; *p* = 0.02). In addition, 56% of participants (49 of 87) had a monthly income as high as J$10,000 to $20,000 versus 35% of comparisons (60 of 173; *p* = 0.00) [27,28]. 

In addition to support of the WCJF efforts, Jamaican legislation was implemented in September of 2013 to increase access to educational institutions for adolescent mothers. The Ministry of Education introduced the ‘Policy on the Re-Integration of Adolescent Mothers into the Formal Education system’, a policy supported by the United Nations Population Fund (UNFPA), which permits adolescents to be readmitted into their high schools following the birth of their babies or attend a new school [22]. UNFPA has funded computer and internet access to the high schools that adolescent mothers attend in order to strengthen the capacity to graduate technologically skilled teens [15]. At the time of the interviews in this study, the adolescents verbalized a desire to return to school, however the policy had not yet been implemented and the discovery of pregnancy was met with expulsion from school. Adolescent mothers were essentially barred from returning to their original schools following birth for fear of the ‘negative influence’ they might have on their peers. They were often hesitant to register in a new school, which often required that they commute to a new neighbourhood. Research will be necessary to explore how well the new policy is being followed by local high school principals. In addition, evaluation of the teen mothers’ perceptions of access and autonomy should be conducted. 

In the adolescent-centred clinic, the participants found less distractions and a more conducive environment to prepare for parenting despite the long waits. Previous research has explored the utility of the “Centering Pregnancy” model [29,30], social media or texting [31] and integrated multidisciplinary [32] approaches in order to improve clinic attendance and meet the learning and social needs of vulnerable populations such as adolescents. Further research is needed to identify if a teen-centred approach also mitigates the effects of psychosocial stress. 

## 5. Conclusions

Despite concerted efforts by the National Centre for Youth Development, the Child Development Agency, local non-governmental organizations and women’s crisis centres, the lived experiences of adolescents are rarely sought. Adolescent pregnancy and birth rates in Jamaica remain one of the highest in the Caribbean [23]. Although motherhood is valued, most of these pregnancies are unplanned by the mother and one in five adolescent girls aged 15–19 experiences forced sexual activity [10]. This is the post-colonial, critical human rights arena from which this study was designed. 

Many Jamaican adolescent mothers face barriers to education, self determination, and family planning. Since adolescence is a vulnerable time in the life-span when there may be readiness to learn; empowering and respectful adolescent-centred health care and comprehensive reproductive health education may mitigate psychosocial distress. The small sample size, urban setting and lack of socio-demographic variable controls prevent the findings from being generalized to the entire population of Jamaican adolescent mothers. The study should be followed by a national school-based survey to further examine the questions that arose concerning the prevalence of psychological distress. Likewise the factors that motivate or deter school completion should be examined. International community-based partnerships with academia, policy-makers, government agencies, service providers and advocates should be formed to create a concerted effort to empower Jamaican youth. Furthermore, the development of new and innovative models of service delivery is necessary. These interventions need to be adapted to low-resourced countries such as Jamaica. Such community-based research, by definition would support resiliency, agency, human rights and access for adolescent mothers in the region. 
